# CircUGP2 inhibits tumor progression via PI3K/AKT/mTOR signaling through sponging miR-137-3p and interacting with UPF1 in hepatocellular carcinoma

**DOI:** 10.3389/fimmu.2026.1926747

**Published:** 2026-10-02

**Authors:** Longfei Xie, Suxin Li, Huahu Guo, Wenhui Zhang, Mengjie Shang, Bailin Cheng, Dingyang Li, Luhao Li, Zhaochen Liu, Lin Li, Liang Bao, Kaiwei Tian, Chengxuan Li, Xiaowei Dang

**Affiliations:** 1 Department of Hepatopancreatobiliary Surgery, The First Affiliated Hospital of Zhengzhou University, Zhengzhou, China; 2 Key Laboratory of Precision Diagnosis and Treatment in General Surgical (Hepatobiliary and Pancreatic) Diseases of Health Commission of Henan Province, Zhengzhou, China; 3 Henan Province Engineering Research Center of Minimally Invasive Diagnosis and Treatment of Hepatobiliary and Pancreatic Diseases, Zhengzhou, China; 4 Budd-Chiari Syndrome Diagnosis and Treatment Center of Henan Province, Zhengzhou, China

**Keywords:** circUGP2, hepatocellular carcinoma, KLF4, PI3K/Akt/mTOR pathway, UPF1

## Abstract

**Background:**

Circular RNA (circRNA) has been shown to have a pivotal role in the initiation and evolution of multiple tumors, but its function in hepatocellular carcinoma (HCC) requires further elucidation.

**Methods:**

circUGP2 (hsa_circ_0001020) was identified in the GEO database by HCC circRNA microarray. Subsequently, an assessment was conducted to determine the biological effects of circUGP2, employing a series of analytical methods, including CCK8, EdU, colony formation, transwell, scratch assays, and *in vivo* experimentation. To explore the functions of circUGP2 as a sponge, including RNA pull-down, microRNA-seq, fluorescence *in situ* hybridization (FISH), RNA immunoprecipitation (RIP), and luciferase reporter assays were employed. Mass spectrometry (MS), RIP, and co-inmunoprecipitation (Co-IP) were employed to examine the interaction between cirrcUGP2 and up-frameshift protein 1 (UPF1).

**Results:**

circUGP2 was downregulated in HCC tissues and cells, which inhibited the growth and spread of HCC *in vitro* and *in vivo*. In the mechanisms, circUGP2 has the capacity to modulate KLF4 by sponging miR-137-3p, thereby suppressing HCC growth and migration. In addition, circUGP2 could combine with UPF1 and facilitate an interplay between UPF1 and AKT, influencing tumor progression in HCC via inactivation of the PI3K/AKT signaling pathway.

**Conclusion:**

circUGP2 has a crucial function in HCC development by modulating the miR-137-3p/KLF4 axis to inactivate the PI3K/AKT/mTOR pathway and by interacting with UPF1. The findings indicate the potential of circUGP2 as a novel biomarker and target for HCC.

## Introduction

Liver cancer poses a major challenge worldwide, imposing a heavy burden on human lives and socioeconomic systems. It is the sixth most prevalent cancer worldwide and the third main contributor to cancer-related fatalities, with incidence rates continuing to rise steadily (1). Although substantial progress has been made in the management and cure of liver cancer, it continues to be a major cause of mortality on a global scale (2). Hepatocellular carcinoma (HCC) represents the most predominant histological form of liver cancer, responsible for 80-90% of liver cancer diagnoses (3). Patients with an early-stage diagnosis of HCC can achieve favorable therapeutic outcomes through approaches such as surgery, ablation, and transplantation. However, early-stage patients often exhibit minimal symptoms and an insidious onset, leading to most cases being detected at an advanced stage where effective treatment is no longer feasible, and the prognosis is poor. For patients in advanced stages, targeted therapies and immunotherapies represent effective treatment options for inhibiting tumor growth (4). Nonetheless, the adverse effects and chemoresistance associated with these medications extend the overall survival of HCC patients by a limited number of months (5). Moreover, the majority of patients remain unresponsive to these treatments, suggesting that most sufferers of advanced HCC cannot obtain effective treatment from the available therapeutic options (6). Consequently, there is an imperative to explore novel treatment methods to address the current situation.

In recent years, insights into the importance of RNA in human biology and illness development have led to novel approaches for cancer treatment. Among these, the function of non-coding RNA (ncRNA) has garnered particular attention. The transcripts of these molecules are evolutionarily conserved and play crucial roles in modulating cellular and metabolic processes (7). Circular RNAs (circRNAs), as a burgeoning type of non-coding RNAs, hold promising potential for application. Numerous studies have demonstrated that the regulatory mechanisms of circRNAs have significant value in both diagnosis and treatment (8). A mounting body of research has revealed that circRNAs are dysregulated in various tumors and can be secreted into bodily fluids, suggesting their potential as promising biomarkers (9, 10). Initially, circRNAs were considered non-functional transcriptional byproducts. However, as research progressed, this previously obscure class of molecules gradually came into focus. Multiple studies have demonstrated their involvement in cellular regulation and molecular mechanisms, and they are also inextricably tied to the growth and progression of liver cancer (11). Recently, owing to the advancement of high-throughput sequencing technology, the expression profiles of circRNAs in various tumors have been extensively mapped, and their functions have gradually been elucidated. These mechanisms primarily include: competitively binding to microRNAs (miRNAs) to regulate gene expression, interacting with RBPs, regulating transcription and translation processes, and even encoding proteins (12, 13). Because of their stability and specificity, circRNAs have notable promise as biomarkers for tumor diagnosis and prognosis (14). Additionally, circRNAs can function as protein scaffolds. Yang et al. (15) discovered that circCCNY acts as a molecular scaffold to promote binding of HSP60 and E3 ubiquitin ligase SMURF1, ultimately inactivating the MAPK signaling pathway. CircRNAs regulate biological processes through functions such as binding to miRNAs and RBPs and translating into peptide chains, demonstrating their immense potential for development.

Research on the circUGP2 molecule remains limited, and its mechanisms in tumors require further investigation. Sunagawa et al. (16) indicates that circRNAs represent highly promising tumor-associated biomarker molecules. They identified three circRNA molecules that are significantly downregulated in HCC and closely associated with patient prognosis, with circUGP2 being one of these three. These findings illustrate the potential research value of circUGP2 and suggest its promise as a new target and biomarker. Yan et al. (17) found that circUGP2 is intimately related to the progression of gastric cancer and significantly correlates with patient prognosis, making it a promising biomarker. These findings indicate its important role in tumors. A study recently showed that circUGP2 is downregulated in cholangiocarcinoma and is closely correlated with prognosis, while the molecular mechanisms involved were also investigated (18). These findings demonstrate that circUGP2 also plays a pivotal role in HCC and participates in regulating key signaling pathways that impact the progression of liver cancer.

In the present study, we identified a circRNA, circUGP2 (hsa_circ_0001020), in the GEO database that is downregulated in HCC tissue and cells by qRT-PCR. Subsequently, we validated the *in vitro* and vivo inhibitory effects of circUGP2 on proliferation and metastasis in HCC. In addition, we proved that circUGP2 functioned as a sponge for miR-137-3p, thereby regulating KLF4 and consequently suppressing PI3K/AKT/mTOR pathway activity. Moreover, our findings indicate that circUGP2 may interplay with UPF1, augmenting the association between UPF1 and AKT, which is required for inactivating the PI3K/AKT signaling pathway.

## Materials and methods

### Patients and clinical samples

The tissue samples were collected from 53 HCC patients who were subjected to surgical procedures between June 2020 and October 2023 at The First Affiliated Hospital of Zhengzhou University. After collection of the samples were rapidly submerged in liquid nitrogen and maintained at -80 °C for prolonged storage. A comprehensive examination of the clinical and pathological data of all enrolled patients was conducted by reviewing their electronic medical records.

### Bioinformatics analysis

The datasets used were accessed from the Gene Expression Omnibus (GEO, https://www.ncbi.nlm.nih.gov/geo) database. Two datasets were retrieved: GSE121714 and GSE155949. Both datasets were derived from the same chip analysis platform, GPL21825. The GEO platform tool GEO2R (https://www.ncbi.nlm.nih.gov/geo/geo2r/) performs preprocessing after the dataset is grouped. The two datasets were divided into three groups for analysis: GSE121714 group (10 non-tumor vs 10 non-metastatic HCC), GSE121714-M group (10 non-metastatic HCC vs 10 metastatic HCC), and GSE155949 group (49 HCC vs 49 peritumoral). After the differentially expressed circRNA data from the three groups were downloaded, the circRNAs were further filtered using the thresholds |logFC| > 1 and *P* < 0.05. Subsequent data analysis was conducted using R software (4.2.2) (https://www.r-project.org/).

The differential expression data of miRNAs in liver cancer and adjacent normal tissues were downloaded from the TCGA dataset via the dbDEMC database (https://www.biosino.org/dbDEMC/). The starBase (https://rnasysu.com/encori/) database was used to predict target miRNAs. The target genes of miR-137-3p were predicted via the miRNet (https://www.mirnet.ca/), miRDB (https://mirdb.org/), and TargetScan (https://www.targetscan.org/vert_80/) databases. PPI interaction networks were predicted via the STRING (https://cn.string-db.org/) database and then imported into Cytoscape (3.9.1) for further screening. The KEGG enrichment analysis bubble plot was generated via R software (4.2.2). The three-dimensional molecular docking model was predicted via the AlphaFold3 online tool (https://alphafoldserver.com/) and analyzed and adjusted via PyMOL (version 3.1) software. The RBPs for circUGP2 were predicted from the starBase, CircNet (https://awi.cuhk.edu.cn/~CircNet/php/index.php), and catRAPID (http://service.tartaglialab.com/page/catrapid_group) databases. RPISeq (http://pridb.gdcb.iastate.edu/RPISeq/) is an online tool that predicts the possibility of binding.

### RNA and gDNA extraction

Total RNA was extracted from samples utilizing Trizol (Invitrogen, USA) in accordance with the accompanying protocol. Genomic DNA (gDNA) was isolated via the genomic DNA extraction kit (Solarbio, China). The concentration of total RNA and DNA was detected utilizing the NanoDrop One (Thermo Scientific, USA).

### Reverse transcription PCR and real-time quantitative PCR

HiScript III All-in-one RT SuperMix Kit (Vazyme, China) was employed for reverse transcription. Subsequently, qRT-PCR was conducted with ChamQ Universal SYBR qPCR Mix (Vazyme, China) on the QuantStudio 5 (Applied Biosystems, USA). The relative RNA expression was calculated by the 2^‐ΔΔCt^ formula and used GAPDH or U6 as internal references. The design of divergent and convergent primers for circUGP2 was undertaken with the objective of facilitating the amplification of circRNA from both complementary DNA (cDNA) and gDNA. The subsequent characterization of the PCR products was conducted through the utilization of 2% agarose gel electrophoresis and Sanger sequencing. The primer sequences are included in **Supplementary Table S2**.

### Cell lines and cell culture

Human HCC cell lines (MHCC-97H, HCC-LM3, HepG2, Huh7, Hep3B, SK-Hep-1, and Li7) and the human embryonic kidney cell line (HEK-293T) were accessed from the Chinese Academy of Sciences. Li7 cells were cultured in RPMI 1640 (Gibco, USA). MHCC-97H, HCC-LM3, HepG2, Huh7, SK-Hep-1, and HEK-293T cells were cultivated in DMEM (Gibco, USA). Hep3B cells were maintained in MEM (Gibco, USA). The incubation environment was 37 °C with 5% CO_2_.

### RNase R assay

The isolated RNA from MHCC-97H and HCC-LM3 cells was separated into two groups. In the experimental group, 2 U RNase R per 1 µg of RNA (Geneseed, China), whereas in the control group, was added to RNase-free water without RNase treatment. Then, qRT-PCR was used to examine the expression of circUGP2 and UGP2.

### Actinomycin D assay

MHCC-97H and HCC-LM3 cells were seeded into a 6-well plate (3×10^5^ cells/well^)^ overnight. The following day, the cells were exposed to 2 µg/ml actinomycin D (MCE, USA) for 4, 8, 12, and 24 h. The relative expression of circUGP2 and UGP2 was measured using qRT–PCR.

### Agarose gel electrophoresis

Prepare a 2% agarose gel and stain with GelstainRed (Uelandy, China). The samples were added to each well and set to 100 V for 1 h. Then, gel images were captured utilizing the FUSION FX Imaging System (Vilber, France).

### Fluorescence *in situ* hybridization assay

FISH probes for circUGP2 and miR-137-3p were constructed by RioBio (China), and FISH was conducted with the Fluorescent *In Situ* Hybridization Kit (RioBio, China). The samples were fixed and then added probes, incubated overnight at 37 °C. Stained cell nuclei using DAPI, and images were acquired with the LSM980 Airyscan2 (Zeiss, Germany).

### Plasmid construction and shRNA interference

For the circUGP2 overexpression plasmids, the human circUGP2 sequence was synthesized and inserted into a CMV-MCS-EF1-ZsGreen1-T2A-puromycin vector (Genechem, China). For the short hairpin RNA (shRNA) assays, three targeting shRNAs and one scrambled shRNA were produced by Genechem (China). The sequences were detailed in **Supplementary Table S2**. Both the overexpression plasmid and shRNAs were transfected via Lipofectamine 3000 (Invitrogen, USA). For stably transfected cell lines, cells were transfected with lentiviruses 3 days and continuously screened with 10 μg/mL puromycin for 5 days.

The synthesis of KLF4 small interfering RNAs (siRNAs) and miR-137-3p mimics was performed by GenePharma (China), and Lipofectamine 3000 (Invitrogen, US) was employed for transfection. The sequences of the siRNAs and mimics are detailed in **Supplementary Table S2**.

### CCK8 assay

The CCK8 assay kit (Dojindo, Japan) was applied to detect cell proliferation potential. Before the initiation of the experiment, the cells were seeded into a 96-well plate (5×10^3^ cells/well). Thereafter, 10 μl CCK8 reagent was pipetted into every well daily. Following 2 h cultivation, the absorbance at 450 nm was determined by the Multi-Mode Microplate Reader (Molecular Devices, USA).

### 5-Ethynyl-2’-deoxyuridine assay

The EdU detection kit (Servicebio, China) was employed for the EdU assay. The cells were seeded into a 24-well plate at 60% density overnight. The following day, 10 μM EdU was pipetted into the medium incubate 2 h. The cells were fixed and permeabilized, and then stained with iFluor 594 reaction mixture for 20 min and Hoechst 33342 for 5 min. The photographs were taken via the fluorescence microscope (Olympus, Japan).

### Colony formation assay

The cells were planted in 6-well plates (2×10^3^ cells/well) and cultivated for 12 days. The cells were then subjected to fixation and subsequently stained with crystal violet staining solution (Beyotime, China) for a period of 20 minutes. Finally, a photograph was captured, and the quantity of colonies was counted via ImageJ.

### Transwell migration/invasion assay

Cells (invasion, 5×10^4^ cells/migration, 2×10^4^ cells) were planted in the upper chamber with (invasion) or without (migration) Matrigel (Corning, USA). Subsequently added with a medium comprising 20% FBS in the lower chamber. After 48 h of incubation, the cells were fixed with 4% paraformaldehyde and then stained. After erasing cells on the upper chamber, images were taken with the light microscope (Olympus, Japan). The quantity of migrating cells was measured by ImageJ.

### Wound healing assay

The cells were seeded in 6-well plates (5×10^5^ cells/well). Upon attaining a cell density of between 90%-100%, a linear wound was created utilizing a pipette tip. Wound images were captured via light microscopy (Olympus, Japan). The proportion of the area healed was analyzed by ImageJ.

### Flow cytometry analysis

The cells were cleaned and harvested with 0.25% trypsin. Then add 1 ml 4 °C 75% ethanol and gently mix, place in a -20 °C freezer for overnight fixation. The cell cycle was examined by employing a Cell Cycle and Apoptosis Kit (Uelandy, China). The cells were incubated with 500 μl propidium iodide (PI) mixture for 20 min and then detected via the FACSCelesta flow cytometry (Becton Dickinson, USA).

The cells were harvested with 0.25% trypsin (without EDTA). Apoptosis was measured via the Annexin V‐APC/7-AAD Apoptosis Kit (Elabscience, China). The cells were incubated for 20 min in the dark with 5 μl Annexin V-APC and 7-AAD, and detected via the FACSCelesta flow cytometry (Becton Dickinson, USA).

### Animal experiments

Female nude mice, 4 weeks old, were obtained from the Animal Research Center of Zhengzhou University. For the xenograft model, 100 µl suspensions comprising 5×10^6^ stable cells (MHCC-97H) were subcutaneously injected into the axilla. We evaluated and recorded tumor size every 5 days. After a month, all mice were euthanized by cervical dislocation. The volume of each tumor was calculated using the equation V = (width^2^ × length)/2, and the weight of tumors was also recorded. The collected tumor tissue samples were divided into two portions. One was promptly frozen in liquid nitrogen for subsequent RNA isolation, while the other was placed in 4% formalin for hematoxylin-eosin (HE) staining and immunohistochemistry (IHC).

For the lung metastasis model, 5×10^6^ stable cells (MHCC-97H) suspended in 100 µl PBS were inoculated into the nude mice via the tail vein. After 6 weeks, live imaging was performed on the nude mice under 3% isoflurane anesthesia, and the mice were subsequently euthanized by cervical dislocation. Lung and liver tissues were harvested for HE staining, and metastatic tumor nodules were examined under a microscope.

### CircUGP2 pull-down assay

An MS2 tag was integrated into the circUGP2 sequence, then the circUGP2‐MS2 overexpression vectors were constructed with pLC5‐ciR (Geneseed, China). After verifying the overexpression efficiency, circUGP2‐MS2 overexpression vectors were co-transfected into HEK-293T cells with the MS2 capture protein, and then pull-down assays were conducted. Finally, the circUGP2 pulldown complexes were identified via miRNA sequencing and mass spectrometry (MS).

### RNA immunoprecipitation

RIP assays were conducted utilizing the RNA Immunoprecipitation Kit (Geneseed, China) according to the provided protocols. The antibodies were mixed with magnetic beads at 4 °C for 2 h. Subsequently, the compounds were mixed with lysates from MHCC-97H and HCC-LM3 cells at 4 °C overnight. The following day, beads were eluted and obtained bound RNAs.

### Co-immunoprecipitation assay

Co-IP assays were conducted utilizing the Co-immunoprecipitation Kit (Geneseed, China) following the provided protocols. Protein complexes were captured using anti-UPF1 or anti-AKT antibodies and IgG at 4 °C overnight. After being mixed with magnetic beads for 2 h, the supernatant was discarded, and the antigen-antibody complex was obtained for the following experiments.

### Dual-luciferase reporter assay

Wild and mutant circUGP2 and KLF4 were synthesized and integrated into the plasmid pmirGLO (GenePharma, China). HEK-293T cells were planted into 24-well plates and cultured to reach 30% confluence within 24 h. The reporter plasmids and different miRNA mimics or the control were subsequently co-transfected into the cells via Lipofectamine 3000. The activities of luciferase were measured with the Dual-Luciferase Reporter Gene Assay Kit (Beyotime, China) after 48 h.

### Immunofluorescence

After the FISH assay, immunofluorescence was conducted, and the cells were blocked with 5% BSA. Next, add the primary antibody and incubate overnight at 4 °C. The following day, incubate with the fluorescent secondary antibody for 2 h. Finally, stain the nuclei with DAPI. The images were captured with the LSM980 Airyscan2 (Zeiss, Germany).

### Immunohistochemistry

The tissue sections underwent a deparaffinization and rehydration procedure in alcohol before antigen retrieval. Next, the slices were blocked for 30 min, and then were incubated with primary antibodies at 4 °C overnight. The following day, incubated with secondary antibodies for 1 h. Finally, the images were captured using Light microscopy (Olympus, Japan).

### Western blotting

The samples were lysed with RIPA lysis buffer (Beyotime, China). The WB procedure was conducted with the manufacturer’s protocols. The ultra-sensitive enhanced chemiluminescent (ECL) substrate (Tanon, China) was used to analyze the protein concentration. The antibodies are detailed in **Supplementary Table S3**.

### Statistical analysis

The statistics were analyzed via SPSS 26.0 and GraphPad Prism 8.0. The data are expressed as the means ± SDs. For the normally distributed data, t-test was utilized for two continuous variable comparisons, one-way ANOVA followed by Tukey HSD *post hoc* test use for three or more sets. When tests of normality were not applicable, the non-parametric test (Mann-Whitney test) was instead. The correlation between circUGP2 and the clinicopathological features of HCC patients was evaluated via the χ2 test. The experimental data were obtained from three biological replicates. Differences were considered statistically significant when *P < 0.05, **P < 0.01, or ***P < 0.001.

## Results

### Identification of circUGP2 and circUGP2 is downregulated in HCC

Through differential circRNA expression analysis of the data divided into three groups, we identified 137 differentially expressed circRNAs in the GSE121714 group, 252 in the GSE121714-M group, and 50 in the GSE155949 group (**Supplementary Figures S1A, B**). The intersection of the three groups resulted in 0 circRNAs being upregulated and 5 circRNAs being downregulated (**Supplementary Figures S1C, D**). By searching the literature and performing ID conversion, we obtained relevant information on these five circRNAs (**Supplementary Table S1**; **Figure 1**). Among them, only circUGP2 (hsa_circ_0001020) in HCC has not been reported. Therefore, circUGP2 was identified as the research subject for further investigation. The downregulation of circUGP2 expression in the GEO database was further validated in 53 paired HCC tissues (**Figures 1B, C**). In HCC cells, the expression of circUGP2 was also analyzed, and we selected MHCC-97H and HCC-LM3 cells for further study (**Figure 1D**). Furthermore, the assessment of HCC clinical data indicated a substantial correlation between diminished circUGP2 and tumor enlargement and advanced TNM stage (**Table 1**).

**Figure 1:**
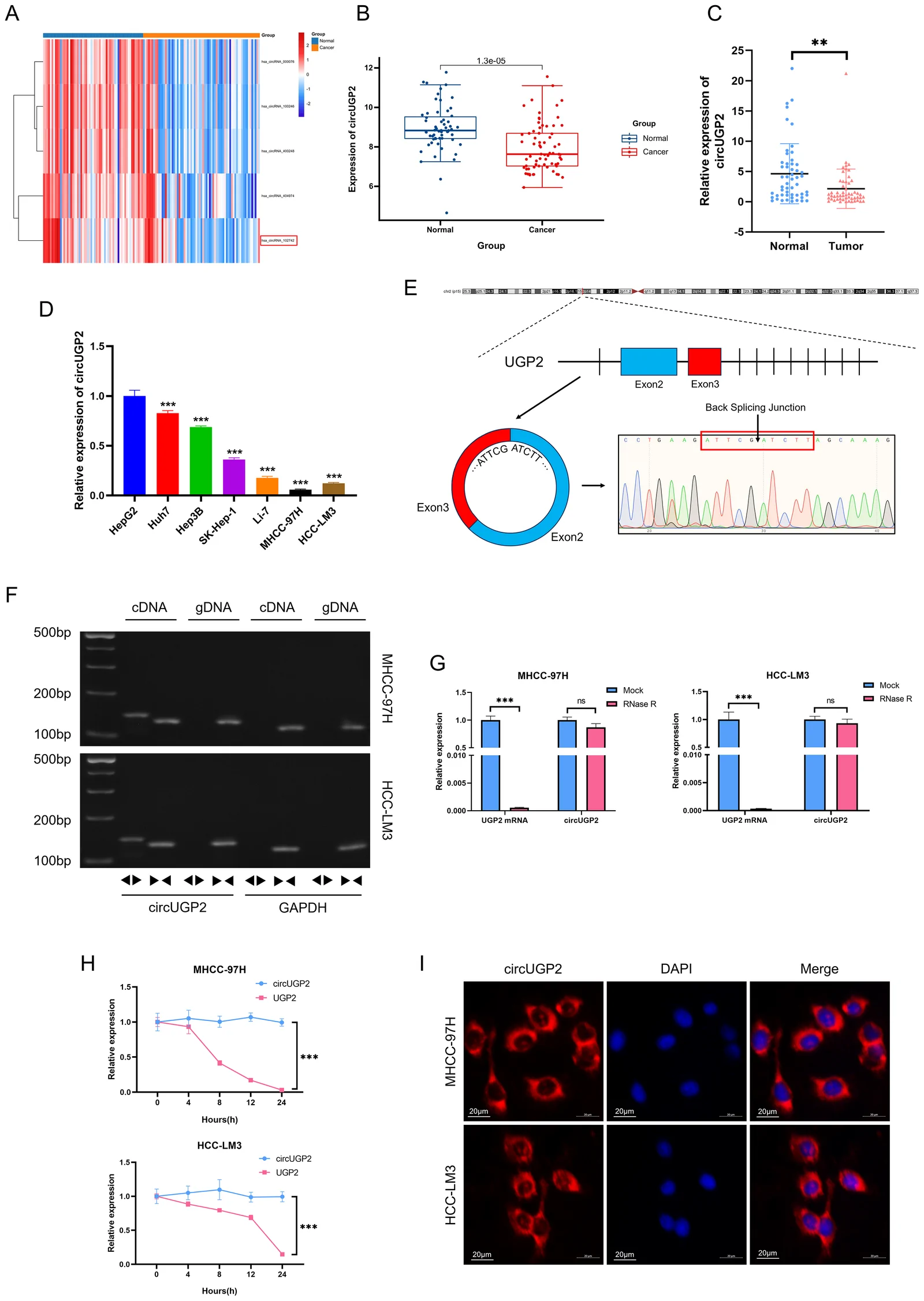
Identification and characteristics of circUGP2 and circUGP2 is downregulated in HCC. **(A)** Heatmap of the 5 co-downregulated differentially expressed circRNAs identified from the GEO database. **(B)** Relative expression levels of circUGP2 in the GEO database. **(C)** Relative mRNA levels of circUGP2 in 53 paired HCC and normal tissues detected by qRT-PCR. **(D)** Relative mRNA levels of circUGP2 in HCC cell lines. **(E)** Schematic illustration of the genomic location and back splicing of circUGP2. Specific divergent primers were designed targeting the back-splice junction, and qRT-PCR products were validated by Sanger sequencing. **(F)** The circular characteristic of circUGP2 was detected by qRT-PCR and agarose gel electrophoresis using divergent and convergent primers in cDNA and gDNA. **(G)** Relative expression levels of circUGP2 and UGP2 mRNA after RNase R treatment. **(H)** The relative expressions of circUGP2 and UGP2 mRNA were detected at the indicated time points after actinomycin D treatment. **(I)** The subcellular location of circUGP2 in HCC cell lines was investigated by FISH assay, scale bar =50 μm. **P < 0.01, ***P < 0.001, ns, no significance.

**Table 1:** Relationship between circUGP2 expression and clinicopathological characteristics in HCC patients.

Characteristics (n=53)	circUGP2 expression		
	Low (n=27)	High (n=26)	*P*-value
Gender			
Male	19 (70.4%)	17 (65.4%)	0.697
Female	8 (29.6%)	9 (34.6%)	
Age(years)			
>60	10 (37.0%)	11 (42.3%)	0.695
≤60	17 (63.0%)	15 (57.7%)	
Tumor differentiation			
G1/G1-G2/G2	18 (66.7%)	12 (46.2%)	0.132
G2-G3/G3	9 (33.3%)	14 (53.8%)	
Tumor diameter			
≥5cm	20 (74.1%)	12 (46.2%)	0.038*
<5cm	7 (25.9%)	14 (53.8%)	
TNM stage			
I-II	11 (40.7%)	19 (73.1%)	0.018*
III-IV	16 (59.3%)	7 (26.9%)	
Nerve invasion			
Yes	3 (11.1%)	5 (19.2%)	0.409
No	24 (88.9%)	21 (80.8%)	
Glisson capsule invasion			
Yes	8 (29.6%)	3 (11.5%)	0.104
No	19 (70.4%)	23 (88.5%)	
Microvascular invasion			
Yes	14 (51.9%)	12 (46.2%)	0.678
No	13 (48.1%)	14 (53.8%)	

Bold values indicates statistical significance, *P<0.05.

CircUGP2 originates from exons 2 and 3 of the UGP2 gene, and its circular structure was validated via Sanger sequencing (**Figure 1E**). Agarose gel electrophoresis showed that circUGP2 was only amplified from cDNA via divergent primers (**Figure 1F**). Furthermore, the findings from the RNase R and actinomycin D assays suggested that circUGP2 showed more stability compared to linear UGP2 (**Figures 1G, H**). CircUGP2 was mainly localized in the cytoplasm of MHCC-97H and HCC-LM3 cells by FISH (**Figure 1I**).

### CircUGP2 inhibits the proliferation and migration of HCC cells *in vitro*

To explore the biological function of circUGP2 in HCC, we designed a plasmid for overexpression, as well as 3 shRNAs targeting the back-splicing junction for the knockdown of circUGP2 in MHCC-97H and HCC-LM3 cells. The efficiency of the overexpression and knockdown of circUGP2 was validated by qRT-PCR (**Figures 2A**, **3A**).

**Figure 2:**
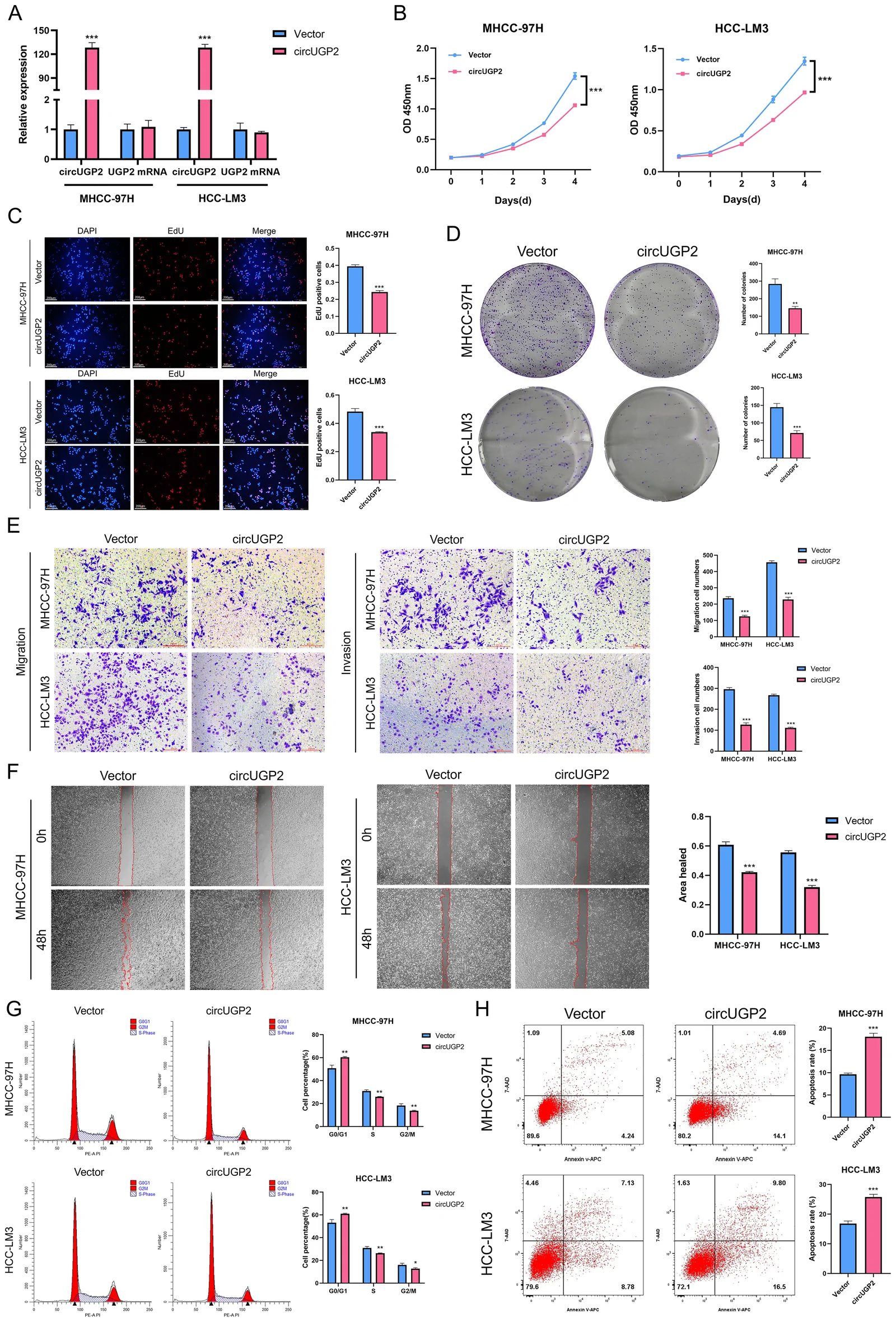
Overexpression of CircUGP2 inhibits the proliferation, migration, and invasion of HCC *in vitro*. **(A)** The expression of circUGP2 and UGP2 mRNA in HCC lines transfected with the plasmid was certified by qRT-PCR. **(B)** CCK8 assay was performed in MHCC-97H and HCC-LM3 cells transfected with the circUGP2 plasmid. **(C)** EdU assay was conducted to evaluate the proliferation in MHCC-97H and HCC-LM3 cells, scale bar =200 μm. **(D)** Colony formation assay of MHCC-97H and HCC-LM3 cells transfected with the circUGP2 plasmid. **(E)** Transwell assay was performed to evaluate the migration and invasion in MHCC-97H and HCC-LM3 cells transfected with the circUGP2 plasmid, scale bar =200 μm. **(F)** Wound healing assay was used to evaluate the migration in MHCC-97H and HCC-LM3 cells, scale bar =500 μm. **(G)** Flow cytometry was used to detect the cell cycle progression in MHCC-97H and HCC-LM3 cells transfected with the circUGP2 plasmid. **(H)** Flow cytometry was used to detect the cell apoptosis rate in MHCC-97H and HCC-LM3 cells transfected with the circUGP2 plasmid. *P < 0.05, **P < 0.01, ***P < 0.001.

**Figure 3:**
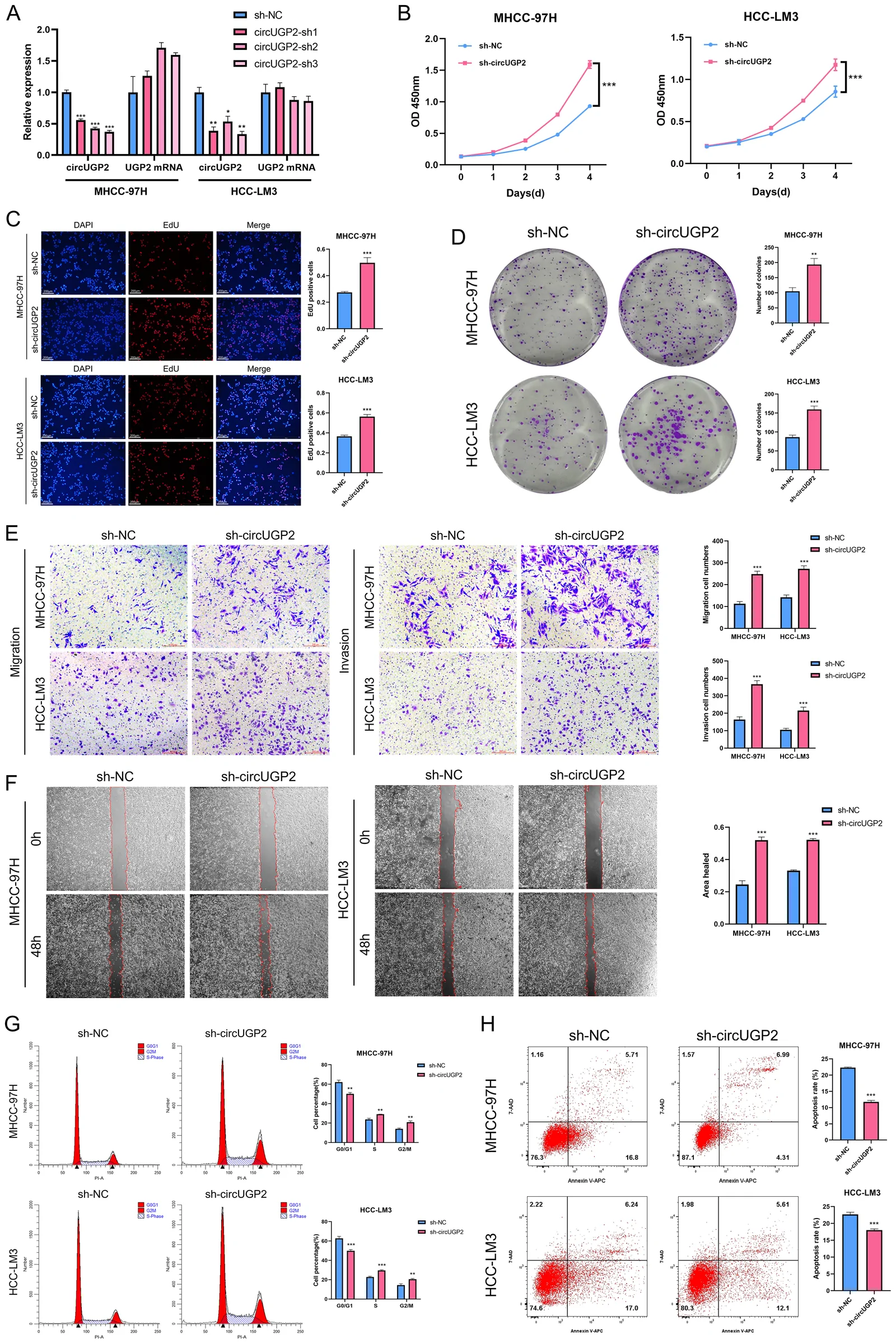
Knockdown of CircUGP2 promotes the proliferation, migration, and invasion of HCC *in vitro*. **(A)** The expression of circUGP2 and UGP2 mRNA in HCC lines transfected with shRNAs was certified by qRT-PCR. **(B)** CCK8 assay was performed in MHCC-97H and HCC-LM3 cells transfected with circUGP2 shRNAs. **(C)** EdU assay was conducted to evaluate the proliferation in MHCC-97H and HCC-LM3 cells, scale bar =200 μm. **(D)** Colony formation assay of MHCC-97H and HCC-LM3 cells transfected with circUGP2 shRNAs. **(E)** Transwell assay was performed to evaluate the migration and invasion in MHCC-97H and HCC-LM3 cells transfected with circUGP2 shRNAs, scale bar =200 μm. **(F)** Wound healing assay was used to evaluate the migration in MHCC-97H and HCC-LM3 cells, scale bar =500 μm. **(G)** Flow cytometry was detected the cell cycle progression in MHCC-97H and HCC-LM3 cells transfected with circUGP2 shRNAs. **(H)** Flow cytometry was detected the cell apoptosis rate in MHCC-97H and HCC-LM3 cells transfected with circUGP2 shRNAs. *P < 0.05, **P < 0.01, ***P < 0.001.

First, the impact of circUGP2 on cell proliferation was detected by CCK8, EdU, and colony formation assays. The results revealed that circUGP2 overexpression suppressed the proliferation of MHCC-97H and HCC-LM3 cells (**Figures 2B–D**). Conversely, the reverse results were shown in the circUGP2 knockdown group (**Figures 3B–D**). Consistently, migration and invasion were hindered by circUGP2 overexpression and enhanced by circUGP2 knockdown, as shown by transwell and wound healing assays (**Figures 2E, F**, **3E, F**). Additionally, flow cytometry analysis revealed that circUGP2 overexpression significantly reduced the proportion of cells in both the S phase (DNA synthesis phase) and the G2/M phase (cell division phase) of the cell cycle (**Figure 2G**). Flow cytometry apoptosis analysis revealed a markedly elevated apoptosis rate in MHCC-97H and HCC-LM3 cells overexpressing circUGP2 (**Figure 2H**). Conversely, the reverse results were shown in the circUGP2 knockdown group (**Figures 3G, H**).

### CircUGP2 inhibits the tumor growth and metastasis of HCC *in vivo*

In the xenograft models, the growth of tumors was notably suppressed by circUGP2 overexpression compared with the control group (**Figures 4A–D**). We simultaneously detected circUGP2 expression levels in mouse tumor tissue and performed HE and immunohistochemistry (IHC) staining. These findings were subsequently corroborated through the detection of Ki-67 levels, as determined by IHC staining (**Figures 4E–G**).

**Figure 4:**
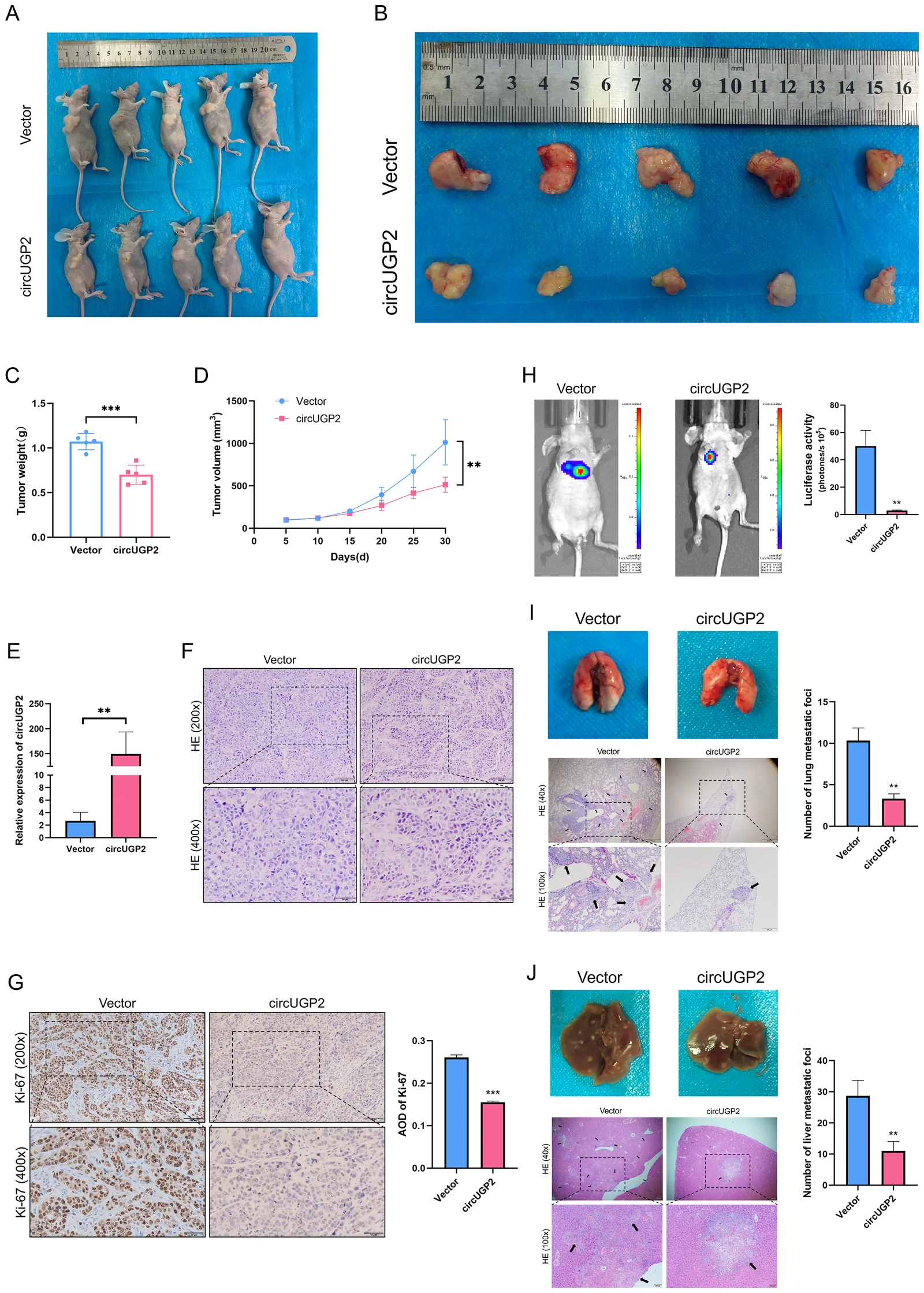
CircUGP2 inhibits the tumor growth and metastasis of HCC *in vivo*. **(A, B)** Representative images of subcutaneous xenograft tumors in nude mice injected with stable circUGP2 overexpression cells (n=5). **(C)** The weight of subcutaneous xenograft tumors shows the effect of circUGP2 overexpression on the formation of HCC. **(D)** Volume of subcutaneous xenograft tumors shows the effect of circUGP2 overexpression on the formation of HCC. **(E)** The expression of circUGP2 in subcutaneous xenograft tumors of circUGP2 overexpression. **(F)** HE staining of subcutaneous xenograft tumors, scale bar =50 μm. **(G)** Ki-67 staining of subcutaneous xenograft tumors, scale bar =100 μm. **(H)** Representative bioluminescence images showed the lung metastasis. **(I)** Metastatic foci from nude mice were shown in lung and HE staining of pulmonary nodules from nude mice, scale bar =500 μm. **(J)** Metastatic foci from nude mice were shown in the liver, and HE staining of liver nodules from nude mice, scale bar =500 μm. **P < 0.01, ***P < 0.001.

In the lung metastasis model. Live imaging revealed that the luciferase luminescence signal intensity in the circUGP2 overexpression group was notably decreased (**Figure 4H**). HE staining revealed that circUGP2 overexpression reduced the count of metastatic nodules in the lung (**Figure 4I**). Interestingly, we detected metastatic lesions not only in the lung tissue of nude mice but also in their liver tissue. These lesions were similarly subjected to HE staining and counting. The staining results revealed that the count of liver metastatic lesions in the circUGP2 overexpressing group was notably decreased (**Figure 4J**).

### CircUGP2 inhibits HCC progression by sponging miR-137-3p

To illuminate the mechanisms by which circUGP2 suppresses HCC development, we employed the MS2-tag system for circUGP2 pull-down assays. First, we conducted miRNA sequencing and proteomic profiling via the pull-down assay with MS2-CP-Flag (**Figure 5A**). The circUGP2-MS2 and MS2-CP-Flag were co-transfected into HEK-293T cells, and qRT-PCR was employed to assess the efficiency (**Figures 5B, C**). Subsequently, an MS2-CP-Flag antibody was employed in conjunction with the capture protein to further enrich for circUGP2 pull-down products. The result of qRT-PCR indicated that circUGP2 was significantly enriched (**Figures 5D, E**).

**Figure 5:**
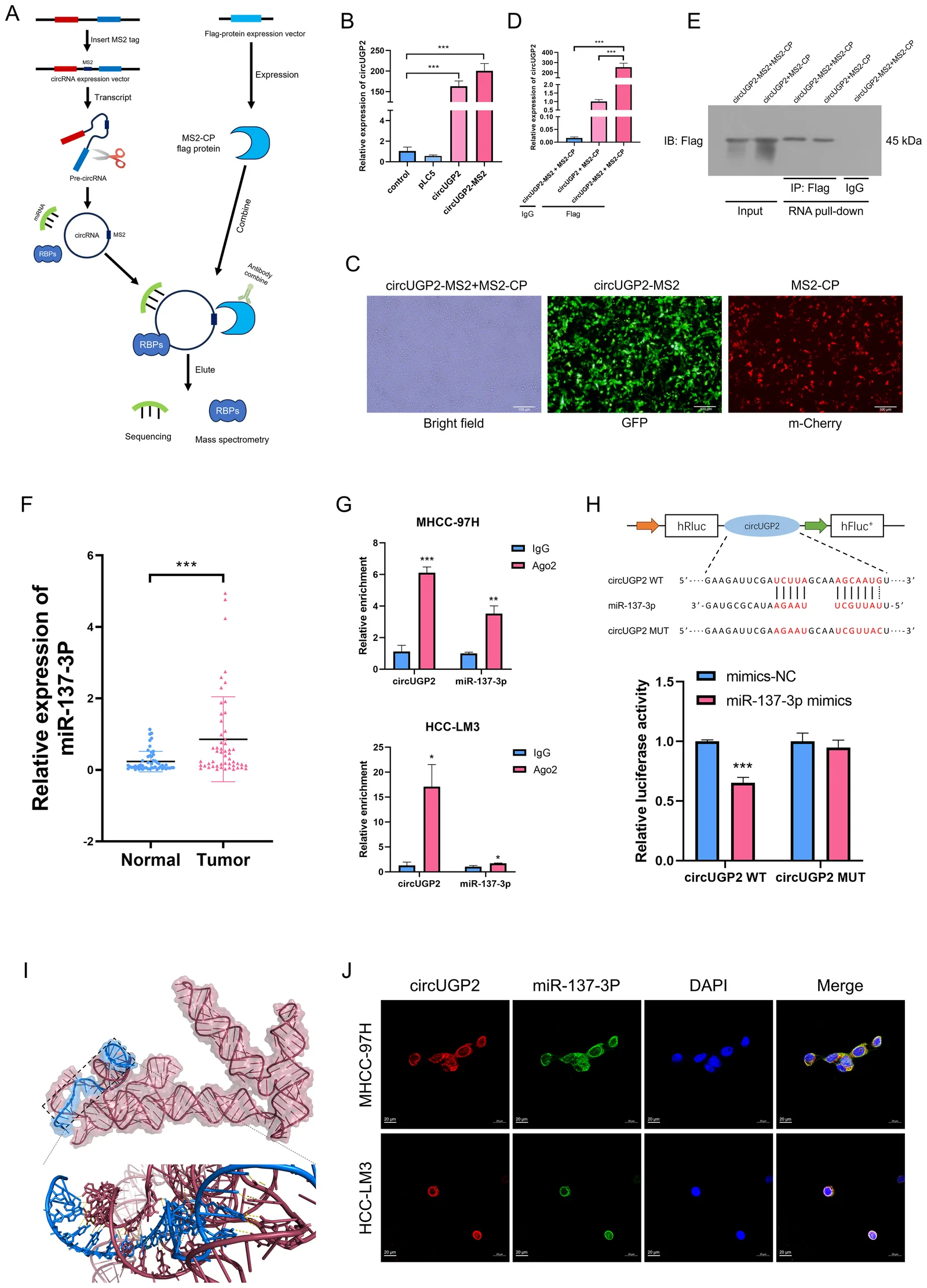
Identification of downstream miRNA molecules by circUGP2 pull-down experiment. **(A)** Schematic illustration of the circUGP2 RNA pull-down assay using the MS2-tagging system. **(B)** Following transfection of the expression plasmids circUGP2, NC (pLC5), and circUGP2-MS2 into HEK-293T cells, the relative expression of circUGP2 was assessed using qRT-PCR. **(C)** Following transfections of MS2-CP and circUGP2-MS2 into HEK-293T cells, fluorescence and bright field images were obtained, scale bar=100 μm. **(D)** The qRT-PCR was used to measure the enrichment of circUGP2 in a complex with MS2-CP-Flag. **(E)** MS2 combined protein in various groups in the RNA pull-down assay was examined using western blotting. **(F)** The expressions of miR-137-3p in 53 paired HCC and normal tissues were detected by qRT-PCR (n=53). **(G)** The interaction between circUGP2 and miR-137-3p was determined by RIP assays using anti-AGO2 or IgG antibodies. **(H)** The wild-type (WT) and mutant (MUT) circUGP2 plasmid for dual-luciferase report were applied to confirm the interaction between circUGP2 and miR-137-3p. **(I)** Schematic diagram of the predicted three-dimensional binding between circUGP2 and miR-137-3p (red represents circUGP2, blue represents miR-137-3p). **(J)** The colocalization of circUGP2 and miR-137-3p was detected by FISH assay, scale bar=20 μm. *P < 0.05, **P < 0.01, ***P < 0.001.

In miRNA-seq, we identified 162 miRNAs that were enriched in the experimental group exclude control group (*P.adj* < 0.05, log|FC|>1). Simultaneously, network regulation visualization analysis was conducted (**Supplementary Figure S2A**). To further screen and identify miRNAs that regulate circUGP2, on the basis of the competing endogenous RNA (ceRNA) mechanism by which miRNAs and circRNAs undergo negative feedback regulation, we retrieved and downloaded miRNAs upregulated in HCC from the TCGA database. By intersecting these with circUGP2 target miRNAs predicted in the starBase database, we identified the target miR-137-3p (**Supplementary Figure S2B**). We subsequently retrieved the expression levels of miR-137-3p in HCC from the starBase database. The results revealed that miR-137-3p was upregulated in HCC tissues and that its overexpression was significantly correlated with patient prognosis (**Supplementary Figures S2C, D**). These results demonstrate that miR-137-3p is a molecule with potential research value and may serve as a target miRNA for circUGP2.

To further confirm the expression level of miR-137-3p in HCC, qRT-PCR analysis of 53 clinical tissue samples revealed significantly higher miR-137-3p expression in HCC tissues than in adjacent tissues (**Figure 5F**). In the MHCC-97H and HCC-LM3 cell lines in which circUGP2 was overexpressed or knocked down, miR-137-3p expression decreased upon circUGP2 overexpression but increased upon circUGP2 knockdown, indicating a negative feedback regulatory relationship between circUGP2 and miR-137-3p (**Supplementary Figures S2E, F**). We constructed miR-137-3p mimics to explore the association between circUGP2 and miR-137-3p (**Supplementary Figure S2G**). The RIP assays revealed that both circUGP2 and miR-137-3p were immunoprecipitated by anti-Argonaute2 (AGO2) in MHCC-97H and HCC-LM3 cells (**Figure 5G**). In addition, we constructed a mutant plasmid for circUGP2, and the results of dual-luciferase reporter assays revealed that luciferase activity was markedly lower in the cells co-transfected with wild-type circUGP2 and the miR-137-3p mimics than in the control group, whereas no significant change was observed in the mutant circUGP2 group (**Figure 5H**). The results of the FISH assays indicated the co-localization of circUGP2 and miR-137-3p in the cytoplasm of MHCC-97H and HCC-LM3 cells (**Figures 5I, J**). These data revealed that circUGP2 acts as a sponge for miR-137-3p in HCC.

To explore the relationship between circUGP2 and miR-137-3p in HCC progression, circUGP2 overexpression vectors and miR-137-3p mimics were co-transfected into MHCC-97H and HCC-LM3 cells, as well as the corresponding control groups. The results of the CCK8, EdU, colony formation, transwell, and wound healing assays showed that the miR-137-3p mimics promoted HCC progression; however, these effects were subsequently partially reversed by circUGP2 overexpression (**Figures 6A–F**).

**Figure 6:**
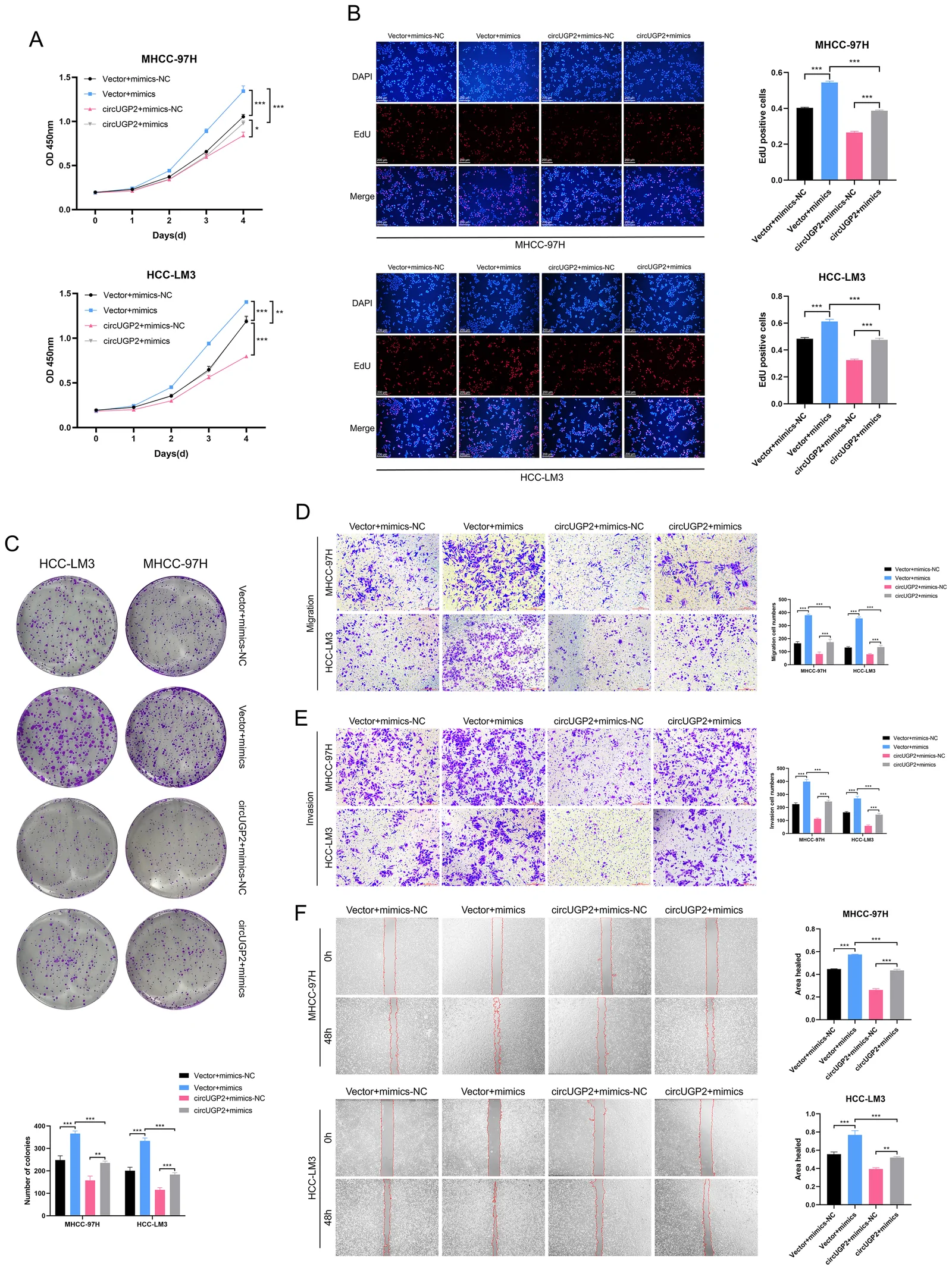
CircUGP2 influences the biological functions of HCC through interaction with miR-137-3p. **(A)** CCK8 assays were performed in circUGP2 overexpression MHCC-97H and HCC-LM3 cells treated with miR-137-3p mimics, as well as the corresponding control groups. **(B)** EdU assays performed in MHCC-97H and HCC-LM3 cells with the indicated treatment, scale bar=200 μm. **(C)** Colony formation assays were performed in MHCC-97H and HCC-LM3 cells with the indicated treatment. **(D)** Transwell migration assays performed in MHCC-97H and HCC-LM3 cells with the indicated treatment, scale bar=200 μm. **(E)** Transwell invasion assays performed in MHCC-97H and HCC-LM3 cells with the indicated treatment, scale bar=200 μm. **(F)** Wound healing assays performed in MHCC-97H and HCC-LM3 cells with the indicated treatment, scale bar=500 μm. *P < 0.05, **P < 0.01, ***P < 0.001.

### CircUGP2 inhibits PI3K/AKT/mTOR signaling pathway via miR-137-3p/KLF4 axis in HCC

To further screen the target genes of miR-137-3p, we used miRNet, miRDB, and TargetScan to predict and identify 235 target genes (**Supplementary Figure S3A**). The KEGG enrichment analysis revealed that the mTOR pathway was enriched. The mTOR signaling pathway is a downstream component of the canonical PI3K/AKT signaling pathway, and this finding aligns with the enrichment analysis of target genes predicted by miRNA-seq analysis (**Supplementary Figures S3B, C**). These results indicate that circUGP2 and miR-137-3p are likely closely related to the PI3K/AKT/mTOR pathway. The 235 identified genes were subsequently subjected to further screening to determine downstream targets. These data were input into STRING to generate a protein-protein interaction (PPI) network, which was then imported into Cytoscape software for additional analysis (**Supplementary Figure S3D**). By retrieving the top 20 genes from the UALCAN database and the Kaplan-Meier plotter database, only KLF4 was found to be significantly decreased in HCC, and its low expression was related to poor patient prognosis (**Supplementary Figures S3E, F**). The qRT-PCR results indicate that KLF4 expression increases when circUGP2 is overexpressed, whereas KLF4 expression decreases when circUGP2 is knocked down, suggesting a positive feedback regulatory relationship (**Supplementary Figures S4A, B**). Similarly, in subcutaneous tumor tissue samples from nude mice, KLF4 expression levels were elevated in circUGP2-overexpressing tissue compared with those in the control group (**Supplementary Figure S4C**). KLF4 expression was found to be lower in HCC in 53 paired clinical tissue samples (**Figure 7A**). The dual luciferase reporter assay subsequently demonstrated a binding relationship between KLF4 and miR-137-3p (**Figure 7B**), further demonstrating that KLF4 may serve as a target for both circUGP2 and miR-137-3p.

**Figure 7:**
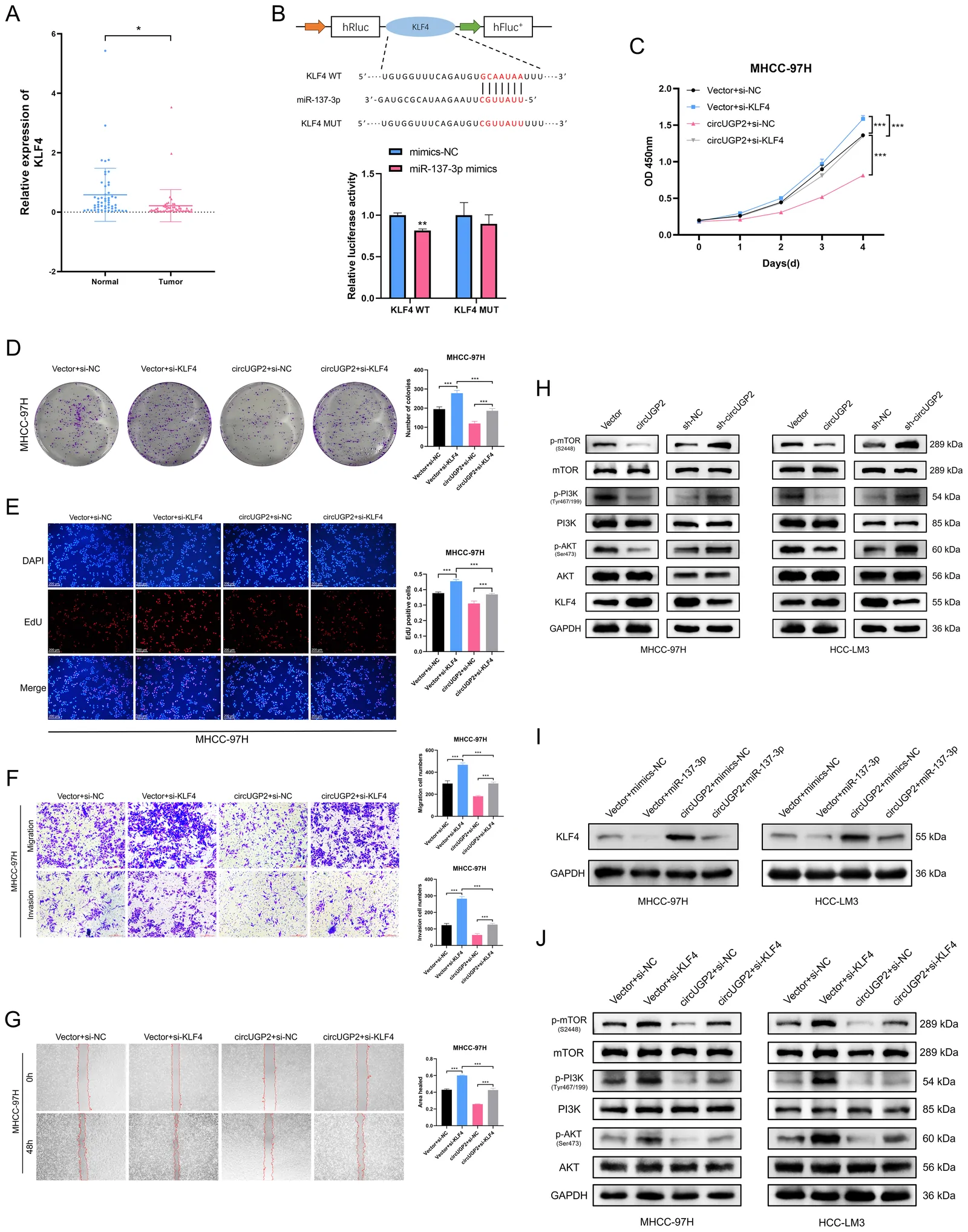
CircUGP2 upregulates KLF4 expression through sponging miR-137-3p and inhibits the PI3K/AKT/mTOR signaling pathway. **(A)** The expression of KLF4 in 53 paired HCC and normal tissues was detected by qRT-PCR (n=53). **(B)** The wild-type (WT) and mutant (MUT) KLF4 plasmid for dual-luciferase report were applied to confirm the interaction between KLF4 and miR-137-3p. **(C)** CCK8 assays were performed in circUGP2 overexpression MHCC-97H cells treated with si-KLF4, as well as the corresponding control groups. **(D)** Colony formation assays performed in MHCC-97H cells with the indicated treatment. **(E)** EdU assays performed in MHCC-97H cells with the indicated treatment, scale bar=200 μm. **(F)** Transwell migration and invasion assays performed in MHCC-97H cells with the indicated treatment, scale bar=200 μm. **(G)** Wound healing assays performed in MHCC-97H cells with the indicated treatment, scale bar=500 μm. **(H)** Western Bolt analysis showed the level of the PI3K/AKT/mTOR signaling pathway in MHCC-97H and HCC-LM3 cells. **(I)** The levels of KLF4 were measured in MHCC-97H and HCC-LM3 cells co-transfected with circUGP2 overexpression and miR-137-3p mimics. **(J)** The levels of PI3K/AKT/mTOR expression were determined by western blot in MHCC-97H and HCC-LM3 cells with the indicated treatment. *P < 0.05, **P < 0.01, ***P < 0.001.

To clarify the relationship between circUGP2 and KLF4, KLF4 was knocked down via small interfering RNAs (siRNAs) targeting KLF4 (**Supplementary Figure S5A**). CircUGP2 overexpression vectors and si-KLF4 were co-transfected into MHCC-97H and HCC-LM3 cells, as were the corresponding control groups. The results of the CCK8, EdU, colony formation, transwell, and wound healing assays showed that KLF4 knockdown promoted HCC progression; however, these effects were subsequently partially reversed by circUGP2 overexpression (**Figures 7C–G**; **Supplementary Figures S5B–F**). Therefore, KLF4 could also be regulated by miR-137- 3p and circUGP2 in HCC. Moreover, the protein level of KLF4 was decreased by the miR-137-3p mimics, which was reversed by circUGP2 overexpression (**Figure 7I**). CircUGP2 can regulate the inactivation of the PI3K/AKT/mTOR pathway in MHCC-97H and HCC-LM3 cells (**Figure 7H**). The protein levels of phosphorylated PI3K/AKT/mTOR were increased by si-KLF4, which was reversed by circUGP2 overexpression (**Figure 7J**). Collectively, these results prove that circUGP2 inhibits the PI3K/AKT/mTOR signaling pathway via the miR-137-3p/KLF4 axis in HCC.

### CircUGP2 interacts with UPF1 to mediate PI3K/AKT signaling pathway

The products from the circUGP2 pull-down assays were further analyzed via MS to investigate the RBP mechanisms. After the control proteins were excluded, a total of 50 proteins were identified as binding partners of circUGP2. To further screen for meaningful proteins that interact with circUGP2, UPF1 proteins were identified by intersecting the predicted RBPs from the starBase, CircNet, and catRAPID databases (**Supplementary Figure S6A**). The RPISeq online tool was used to predict binding ability, and the predicted score indicated a very high probability of circUGP2 binding to UPF1 (**Supplementary Figure S6B**). Subsequent KEGG and Reactome enrichment analyses of the MS results revealed the following mRNA regulatory pathway: nonsense-mediated mRNA decay (NMD). UPF1 is a core protein involved in the NMD mechanism, suggesting that circUGP2 and UPF1 may be closely associated with the NMD (**Supplementary Figures S6C, D**). In silver staining assays, a difference band of around 130 kDa was discovered, and the results of MS indicated that UPF1 may be able to combine with circUGP2 (**Figures 8A, B**). WB assays of pull-down products revealed the presence of UPF1 protein in the circUGP2 group (**Figure 8C**). Subsequently, the interaction between circUGP2 and UPF1 in MHCC-97H and HCC-LM3 cells was verified by RIP assays (**Figure 8D**). We validated the co-localization of circUGP2 and UPF1 by FISH and immunofluorescence (**Figure 8E**). Interestingly, circUGP2 knockdown or overexpression did not affect the mRNA or protein expression of UPF1 (**Figures 8F, G**).

**Figure 8:**
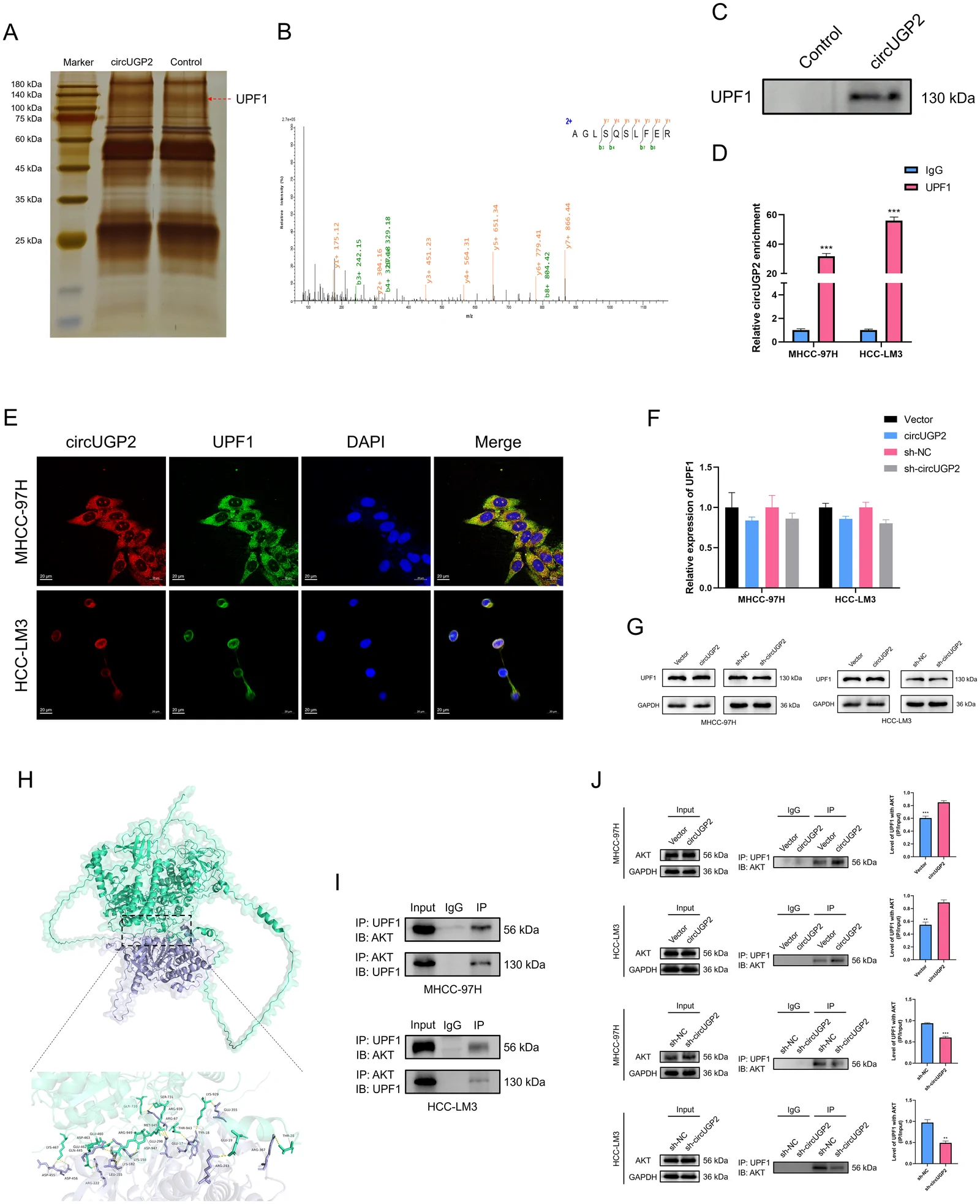
CircUGP2 interacts with UPF1 to mediate PI3K/AKT signaling pathway inactivation. **(A)** Silver staining following RNA pull-down assays. A specific band, indicated by the arrow, appeared at approximately 130 kDa. **(B)** The typical UPF1 peptide was identified in circUGP2-enriched proteins based on MS analysis. **(C)** UPF1 was detected by WB using proteins pulled down by RNA pull-down assays. **(D)** RIP assays were performed using UPF1 or IgG antibodies. **(E)** The co-localization of circUGP2 and UPF1 was detected by FISH assays and immunofluorescence assays, scale bar=20 μm. **(F)** The mRNA levels of UPF1 were detected by qRT-PCR in circUGP2 overexpression or knockdown MHCC-97H and HCC-LM3 cells. **(G)** The protein levels of UPF1 were examined by western blot in circUGP2 overexpression or knockdown MHCC-97H and HCC-LM3 cells. **(H)** Molecular docking 3D model of UPF1 and AKT1 (green represents the UPF1 protein; purple represents the AKT1 protein). **(I)** The binding of UPF1 to AKT was confirmed by co-IP with AKT or UPF1 antibody. **(J)** Co-IP assays were applied to investigate the interaction between UPF1 and AKT in the MHCC-97H and HCC-LM3 cells transfected with circUGP2 overexpression or circUGP2 knockdown. **P < 0.01, ***P < 0.001.

A literature review revealed that UPF1 can bind to AKT1, thereby influencing the NMD mechanism. Another mode of circRNA-protein interaction involves its function as a molecular scaffold. UPF1 and AKT may interact, and this interaction may be regulated by circUGP2. Subsequently, molecular docking between UPF1 and AKT1 was predicted using AlphaFold3 (**Figure 8H**). We hypothesized that circUGP2 may act as a scaffold to promote NMD activation. UPF1 can interact with AKT and is involved in the PI3K/AKT pathway. To test whether UPF1 interacts with AKT in HCC, we performed immunoprecipitation and WB assays in MHCC-97H and HCC-LM3 cells. The results indicated that AKT was co-immunoprecipitated with UPF1, and UPF1 was also co-immunoprecipitated with AKT (**Figure 8I**). To assess whether circUGP2 affects the interaction between UPF1 and AKT in HCC, we conducted immunoprecipitation experiments with UPF1 antibodies in both MHCC-97H and HCC-LM3 cells with circUGP2 overexpression or knockdown. We observed that circUGP2 overexpression promoted the interaction between UPF1 and AKT with no change in protein levels; in contrast, circUGP2 knockdown attenuated the interaction between UPF1 and AKT in HCC cells (**Figure 8J**). These results indicate that circUGP2 can interact with UPF1 and subsequently promote the interaction between UPF1 and AKT in HCC.

## Discussion

Hepatocellular carcinoma (HCC), the predominant histological subtype of liver cancer, poses a significant threat to human health worldwide due to its persistently high incidence and mortality rates. Its complex pathogenesis presents numerous challenges for clinical management (3). Despite continuous advances in surgery, chemotherapy, and targeted therapies, challenges such as difficulties in early diagnosis, high recurrence rates, and multidrug resistance continue to hinder improvements in patient prognosis (19). Therefore, exploration of the molecular mechanisms underlying HCC is highly important for identifying novel diagnostic biomarkers and therapeutic targets. CircRNAs, non-coding RNA molecules with a closed-loop structure, have become a hotspot in tumor biology research due to their rich regulatory functions. CircRNAs exhibit diverse functions, including competitive binding with miRNAs, regulation of RBPs, participation in transcriptional regulation, and even translational coding capability (20). In HCC, circRNAs not only participate in regulating tumor proliferation, migration, invasion, and apoptosis but also play crucial roles in tumor stem cell properties, immune evasion, metabolic reprogramming, and drug resistance (21, 22).

CircUGP2 originates from chromosome 2 (chr2:64083439-64085070), which is formed by reverse splicing and circularization of exons 2 and 3 of the parental gene UGP2 (uridine diphosphate glucose pyrophosphorylase 2), with a total length of 236 bp. In a high-throughput sequencing study of circRNAs in liver cancer patients, seven circRNA candidate molecules were identified, including circUGP2 (hsa_circ_0001020), although its specific molecular mechanism remains unexplained (16). These indicate that circUGP2 is a highly promising research molecule in HCC. In our study, circUGP2 was significantly decreased in HCC tissues. Furthermore, its reduced expression in HCC tissues was notably correlated with tumor size and stage in HCC patients. We constructed circUGP2 overexpression and knockdown plasmids and investigated their effects *in vitro*. These results collectively indicate that circUGP2 may function as a tumor suppressor gene. Cell cycle alterations and apoptosis are inextricably linked to tumor proliferation, often manifesting as dysregulation within tumors. One of the fundamental characteristics of tumor cells is their capacity for unlimited proliferation. The active proliferation of cells frequently accompanies imbalances in cell cycle progression and apoptosis regulation (23, 24). Simultaneously, we examined changes in the cell cycle and apoptosis of HCC cells. We found that circUGP2 inhibits HCC cells from entering the S phase and G2/M phase, thereby reducing their proliferative capacity. Flow cytometry apoptosis assays revealed that circUGP2 overexpression increased the proportion of apoptotic cells, further diminishing HCC cell proliferation. Using subcutaneous tumor models and lung metastasis models in nude mice, we found that circUGP2 overexpression significantly suppressed subcutaneous tumor growth and the formation of lung metastatic lesions.

The miR-137-3p also plays a crucial role in tumor development, and its dysregulation has been confirmed across multiple tumor types. Numerous studies have demonstrated that miR-137-3p is significantly overexpressed in HCC tissues (25, 26). Furthermore, the expression level of miR-137-3p is closely associated with multiple clinical and pathological characteristics, including tumor clinical stage, tumor size, and vascular invasion, in HCC patients (27). These studies indicate that miR-137 may also play a crucial role in HCC. Its abnormal expression levels may influence the biological behavior of HCC cells by regulating specific target genes and signaling pathways. However, the majority of the literature indicates that miR-137-3p acts as a tumor suppressor in gastrointestinal tumors. This discrepancy may suggest that the role of miR-137-3p in HCC is context-dependent, and its function may be regulated by various factors, including the tumor microenvironment, genetic, and molecular subtypes (28). Furthermore, the expression of miR-137-3p is regulated by a complex network of upstream regulatory factors, including transcription factor binding, promoter methylation, and the ceRNA mechanism involving lncRNAs and circRNAs (29, 30). These findings suggest that the expression and function of miR-137-3p in hepatocellular carcinoma may be associated with various factors, such as the tumor microenvironment and molecular background. Future multicenter studies with larger sample sizes and stricter stratification are needed to elucidate its precise role in hepatocellular carcinoma and to explore its clinical value as a diagnostic and prognostic biomarker for the disease.

We obtained complexes that interact with circUGP2 through RNA pull-down experiments and subsequently elucidated their underlying molecular mechanisms via miRNA sequencing and proteomic analysis. Through the identification and analysis of the circUGP2 pull-down complex and further bioinformatics analysis, the target miRNA molecule miR-137-3p was identified. A search of the starBase database revealed its high expression in HCC tissues, with this overexpression significantly correlated with patient prognosis. These results indicate that miR-137-3p may also be a target molecule with potential research value. By detecting miR-137-3p expression levels in our collected clinical tissue samples, we confirmed its statistically significant overexpression in HCC tissues. Subsequent detection of the miR-137-3p expression levels in the circUGP2 overexpression and knockdown groups revealed that the miR-137-3p expression level decreased when circUGP2 was overexpressed and increased when circUGP2 was knocked down compared with the control group, demonstrating a negative feedback regulatory mechanism. Binding experiments, dual luciferase reporter assays, and FISH experiments confirmed their association within the cytoplasm, suggesting significant regulatory implications. Functional recovery experiments further clarified their regulatory relationship, demonstrating a competitive binding interaction between miR-137-3p and circUGP2, confirming its role as a target miRNA for circUGP2.

KLF4 (Kruppel-Like Factor 4), a key member of the Kruppel-like factor family, is a DNA-binding transcription factor that acts as a “molecular switch” in various physiological and pathological processes (31). In tumor-related research, multiple members of the KLF family, including KLF4, have been reported to be associated with tumorigenesis (32). In HCC, KLF4 typically functions as a tumor suppressor gene (33). Extensive clinical studies have demonstrated that KLF4 expression is significantly downregulated in HCC tissues. Furthermore, it serves as an independent prognostic factor for reduced overall survival in patients (34). Targeting KLF4 or its regulatory pathways may offer novel therapeutic approaches for HCC. The natural product curcumin inhibits the transcription factor Sp1 by upregulating KLF4, thereby downregulating the expression of the apoptosis antagonist AATF and delaying the progression of non-alcoholic steatohepatitis to HCC (35). These studies indicate that KLF4 exerts its tumor-suppressive effects through multiple pathways, providing potential therapeutic targets for HCC treatment. We identified 235 potential targets by jointly predicting the target molecules of miR-137-3p across three databases and taking their intersection. KEGG enrichment analysis revealed similar pathways enriched in both the sequencing data target molecule enrichment analysis and the miR-137-3p target analysis: the PI3K/AKT/mTOR pathway. We hypothesize that circUGP2 likely plays a crucial role in the PI3K/AKT/mTOR pathway. Subsequently, the 235 screened target genes were imported into Cytoscape via a PPI network to identify the most closely connected core genes. Analysis of the top 20 most closely related molecules revealed their expression levels in HCC tissues. Survival analysis further identified KLF4 as a core gene whose expression was significantly decreased in HCC and showed a remarkable correlation between low KLF4 expression and poor prognosis in HCC patients. Further validation in clinical tissue samples confirmed lower expression of KLF4 in HCC tissues than in peritumoral tissues. Both its transcriptional and protein levels are regulated by circUGP2, revealing a positive feedback regulatory relationship between circUGP2 and KLF4. To clarify this regulatory interaction, functional recovery experiments were conducted with circUGP2 and KLF4, which validated their mutual regulation. Additionally, circUGP2 was found to regulate the PI3K/AKT/mTOR pathway. The overexpression of circUGP2 led to inactivation of this pathway, whereas its knockdown resulted in pathway activation.

The UPF1 protein (up-frameshift protein 1) is a core protein in the nonsense-mediated mRNA decay (NMD) pathway and plays a crucial role in the initiation and progression of various tumors (36). We demonstrated the binding relationship between circUGP2 and UPF1 through RIP and FISH immunofluorescence experiments, confirming that both primarily co-localize in the cytoplasm. To further explore the relationship between circUGP2 and UPF1, we overexpressed and knocked down circUGP2. The results indicated that the transcriptomic and protein levels of UPF1 remained largely unchanged, suggesting that no regulatory relationship likely exists between circUGP2 and UPF1. A literature review revealed that, in addition to regulatory interactions, circRNAs and RBPs exhibit multiple functional relationships. RBPs are a class of proteins capable of specifically recognizing and binding to RNA, playing a central role in post-transcriptional regulation (37). In HCC, circRNAs interact with RBPs through multiple mechanisms: on the one hand, circRNAs can act as “molecular sponges” to competitively bind RBPs, altering their regulatory capacity over target mRNAs (38); on the other hand, circRNAs can function as scaffold proteins to recruit RBPs into functional complexes or influence their subcellular localization by sequestering RBPs (39). These interactions not only influence the expression of genes but also participate in key processes such as metabolic reprogramming, epithelial-mesenchymal transition (EMT), and tumor microenvironment remodeling in HCC (40, 41). These indicate that circUGP2 may function as a molecular scaffold for cellular regulation.

UPF1, as a core factor in the NMD pathway, also plays a vital role in modulating the PI3K/AKT pathway. The NMD mechanism can specifically recognize and degrade abnormal mRNAs containing premature termination codons caused by upstream open reading frames or intron retention (42). Research has revealed that multiple negative regulators encoding the PI3K/AKT pathway can be degraded by NMD, with the PTEN gene being the most representative example. As a primary negative regulator of the PI3K/AKT pathway, PTEN undergoes stringent regulation via NMD. When UPF1 function is downregulated, the NMD degradation capacity diminishes, leading to abnormal accumulation of PTEN. This interferes with normal PTEN protein expression, thereby indirectly enhancing the activity of the PI3K/AKT pathway (42, 43). Palma et al. (42) indicate that UPF1 can interact with AKT1, promoting UPF1 phosphorylation and thereby initiating the NMD process. This study provides insights for our investigations, suggesting that UPF1 may also bind to AKT1 in HCC cells and potentially promote NMD activation. CircUGP2 likely functions as a “molecular scaffold” in this process. To this end, we validated the interaction between UPF1 and AKT through bidirectional Co-IP experiments. Co-IP conducted during circUGP2 overexpression revealed that UPF1 pulled down more AKT, whereas circUGP2 knockdown resulted in reduced AKT protein levels pulled down by UPF1. These indicate that circUGP2 functions as a molecular scaffold, forming a complex with UPF1 and AKT. The involvement of circUGP2 promotes the binding of UPF1 and AKT. This promotes UPF1 phosphorylation and activates the NMD mechanism. The NMD mechanism rapidly degrades abnormal PTEN transcripts, allowing normal PTEN protein expression and function, thereby indirectly inactivating the PI3K/AKT signaling pathway. UPF1 is an ATP-dependent RNA helicase traditionally considered responsible for recognizing and degrading abnormal mRNAs containing premature termination codons (44). However, recent studies have revealed that UPF1 functions extend far beyond NMD. It can directly bind to the flanking intron regions of circRNAs or mature circRNA molecules, extensively participating in circRNA biogenesis, stability regulation, and functional expression (45, 46). Fischer et al. (47) discovered that UPF1 synergistically interacts with G3BP1 to mediate circRNA degradation by recognizing highly structured RNA regions. These indicate that UPF1 may participate in the degradation process of circRNAs, suggesting a potential novel degradation mechanism targeting circRNAs.

## Conclusion

In conclusion, our study revealed that circUGP2 is downregulated in HCC and functions as a sponge of miR-137-3p, resulting in the downregulation of KLF4, which further activates the PI3K/AKT/mTOR pathway in HCC. In addition, circUGP2 promotes the binding of UPF1 to AKT by acting as a molecular scaffold, thereby facilitating the phosphorylation of the UPF1 protein to activate NMD and subsequently inactivate the PI3K/AKT pathway (**Figure 9**). Overall, our study demonstrated that circUGP2 may serve as a novel biomarker for HCC patients.

**Figure 9:**
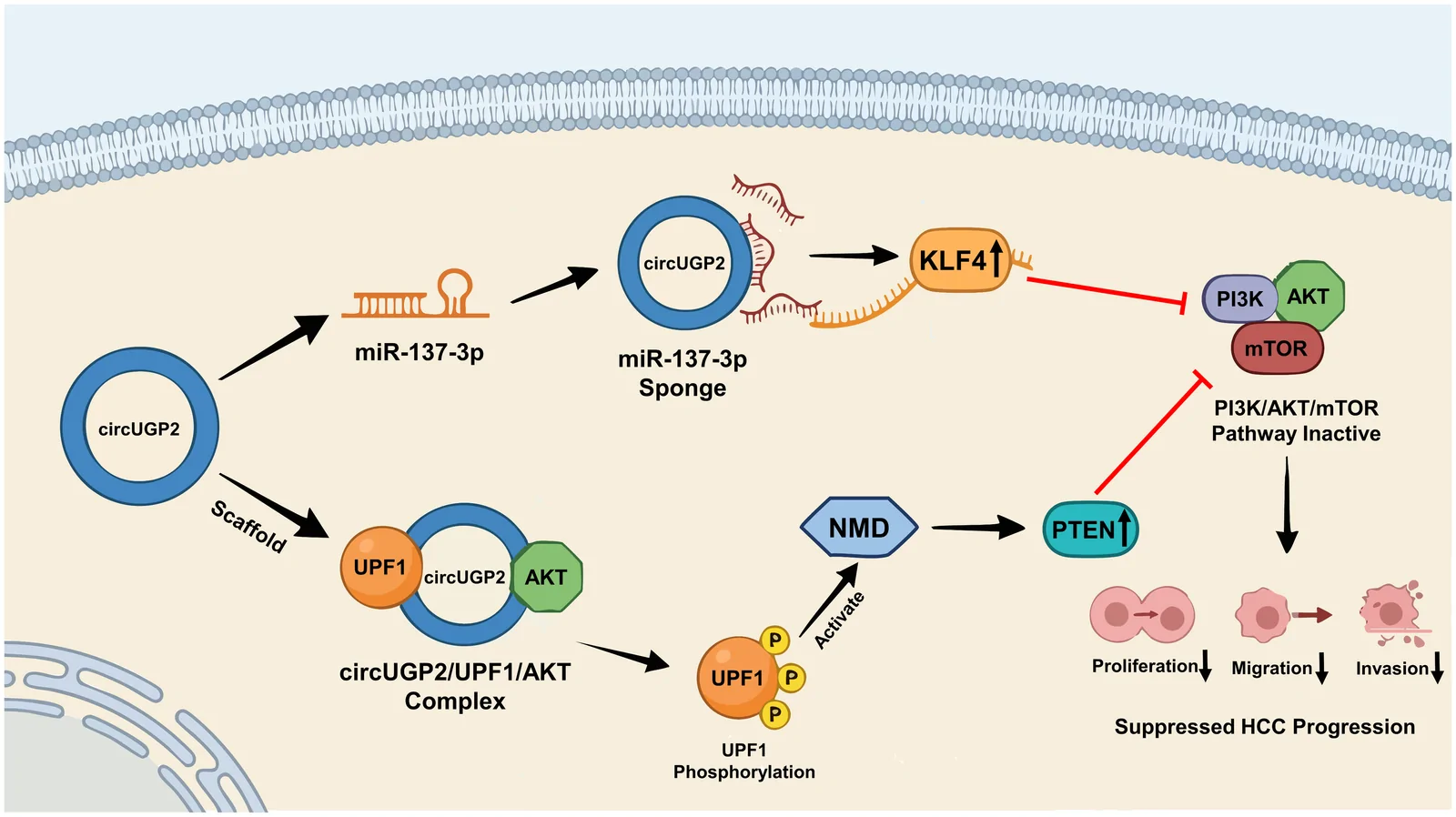
Schematic diagram illustrating the mechanism by which circUGP2 suppressed HCC progression. UPF1, up-frameshift protein 1; NMD, nonsense-mediated mRNA decay; PTEN, phosphatase and tensin homolog.

## Data Availability

The original contributions presented in the study are included in the article/**Supplementary Material**. Further inquiries can be directed to the corresponding author.

## Supplementary material

Supplementary Figure 1 Screening of differentially expressed circRNAs in the GEO database. **(A)** The volcano plot of differentially expressed circRNAs in GEO datasets. **(B)** Heatmap of the differentially expressed circRNAs in GEO datasets. **(C)** A Venn diagram of 3 groups of highly differentially expressed circRNAs was intersected to screen for co-upregulated circRNAs. **(D)** A Venn diagram of 3 groups of lowly differentially expressed circRNAs was intersected to screen for co-downregulated circRNAs.

Supplementary Figure 2 Bioinformatics analysis and validation of downstream miRNA molecules. **(A)** Predicted network regulation diagram of RNA pull-down assays enriched miRNA-targeted mRNAs (showing only the top 300 with the highest scores). **(B)** Venn diagram showing the intersection of miRNA-seq data combined with TCGA HCC miRNA upregulation datasets and starBase predicted target miRNAs. **(C)** The starBase database was used to retrieve the expression levels of miR-137-3p in liver cancer tissues and normal tissues. **(D)** Survival analysis of miR-137-3p in HCC patients using the starbase database. **(E, F)** The expression levels of miR-137-3p in MHCC-97H and HCC-LM3 cells following circUGP2 overexpression and knockdown. **(G)** The expression levels of constructed miR-137-3p mimics/mimics-NC were determined after transfection of MHCC-97H and HCC-LM3 cells. **P* < 0.05, ***P* < 0.01, ****P* < 0.001.

Supplementary Figure 3 Bioinformatics analysis of miR-137-3p downstream target genes. **(A)** Venn diagram showing the intersection of miR-137-3p target genes predicted by miRNet, miRDB, and TargetScan databases. **(B)** KEGG enrichment analysis of miRNA-seq predicted target genes. **(C)** KEGG enrichment analysis of 235 predicted target genes of miR-137-3p. (The red-framed sections indicate enriched similar pathways.) **(D)** The screening process of PPI networks in the Cytoscape software. **(E)** The expression levels of KLF4 in HCC tissues and normal tissues in the UALCAN database. **(F)** Survival analysis of KLF4 in HCC patients in the Kaplan-Meier plotter database.

Supplementary Figure 4 The expression levels of KLF4 in cells and tissues. **(A, B)** The expression levels of KLF4 in MHCC-97H and HCC-LM3 cells following circUGP2 overexpression and knockdown. **(C)** The expression levels of KLF4 in subcutaneous tumor tissue samples from nude mice. **P* < 0.05, ***P* < 0.01, ****P* < 0.001.

Supplementary Figure 5 CircUGP2 upregulates KLF4 expression through sponging miR-137-3p in HCC-LM3 cells. **(A)** The knockdown effects of the 3 designed si-KLF4 in MHCC-97H and HCC-LM3 cells. **(B)** CCK8 assays were performed in circUGP2 overexpression HCC-LM3 cells treated with si-KLF4, as well as the corresponding control groups. **(C)** EdU assays performed in HCC-LM3 cells with the indicated treatment, scale bar=200 μm. **(D)** Colony formation assays were performed in HCC-LM3 cells with the indicated treatment. **(E)** Transwell migration and invasion assays performed in HCC-LM3 cells with the indicated treatment, scale bar=200 μm. **(F)** Wound healing assays performed in HCC-LM3 cells with the indicated treatment, scale bar=500 μm. **P* < 0.05, ***P* < 0.01, ****P* < 0.001.

Supplementary Figure 6 Bioinformatics analysis of circUGP2 RNA-binding proteins. **(A)** Venn diagram of mass spectrometry (MS) results and predictions from starBase, CircNet, and catRAPID databases. **(B)** The RPISeq online tool predicts the binding potential between circUGP2 and the UPF1 protein. (The red box indicates the predicted result score.) **(C)** KEGG enrichment analysis bubble plot. **(D)** Reactome enrichment analysis bubble plot. (The red box indicates enriched analysis of similar pathways.).
