# Supplementary material for: CircUGP2 inhibits tumor progression via PI3K/AKT/mTOR signaling through sponging miR-137-3p and interacting with UPF1 in hepatocellular carcinoma
Source: Front Immunol. 2026 Oct 2;17:1926747. doi: 10.3389/fimmu.2026.1926747 (PMC13672610; doi:10.3389/fimmu.2026.1926747)
Supplement: Supplementary file 1 [file Table1.docx]

**Table S1** circRNAs retrieval information

| CircRNA arraystar ID | Chr | Chr start | Chr end | Gene symbol | Strand | Spliced length | CircBase ID | Reference |
| --- | --- | --- | --- | --- | --- | --- | --- | --- |
| hsa_circRNA_404974 | Chr12 | 23998916 | 24048958 | SOX5 | - | 443 | hsa_circ_0098181 | Reported |
| hsa_circRNA_100246 | Chr1 | 59805629 | 59844509 | FGGY | + | 353 | hsa_circ_0006633 | Reported |
| hsa_circRNA_400248 | Chr1 | 59811918 | 59844509 | FGGY | + | / | Not Found | / |
| hsa_circRNA_000076 | Chr1 | 59805629 | 59812070 | FGGY | + | / | Not Found | / |
| hsa_circRNA_102742 | Chr2 | 64083439 | 64085070 | UGP2 | + | 236 | hsa_circ_0001020 | Not Reported |
