# Supplementary material for: CircUGP2 inhibits tumor progression via PI3K/AKT/mTOR signaling through sponging miR-137-3p and interacting with UPF1 in hepatocellular carcinoma
Source: Front Immunol. 2026 Oct 2;17:1926747. doi: 10.3389/fimmu.2026.1926747 (PMC13672610; doi:10.3389/fimmu.2026.1926747)
Supplement: Supplementary file 2 [file Table2.docx]

**Table S2.** Primers, siRNAs and probes used in this study.

| Name | Sequence (5’→3’) |
| --- | --- |
| CircUGP2 Forward | CCCTGAAGATTCGATCTTAGCAA |
| CircUGP2 Reverse | TGTGATGATGCTGTGGTGAG |
| UGP2 Forward | ATGTCTCAAGATGGTGCTTCTCA |
| UGP2 Reverse | GGTGTGCTCAAATTCATGTGATG |
| GAPDH Forward | TTCGACAGTCAGCCGCATCTTCTT |
| GAPDH Reverse | CAGGCGCCCAATACGACCAAATC |
| GAPDH (Divergent) Forward | TGTACCATCAATAAAGTACCCTGTG |
| GAPDH (Divergent) Reverse | AAATCCGTTGACTCCGACCT |
| CircUGP2 FISH probe | GCTTTGCTAAGATCGAATCTTCAGGG |
| CircUGP2 si-1 | ATTCGATCTTAGCAAAGCAAT |
| CircUGP2 si-2 | CCTGAAGATTCGATCTTAGCA |
| CircUGP2 si-3 | TGAAGATTCGATCTTAGCAAA |
| CircUGP2 si-NC | TTCTCCGAACGTGTCACGT |
| miR-137-3p-RT | GTCGTATCCAGTGCAGGGTCCGAGG  TATTCGCACTGGATACGACATTATC |
| miR-137-3p Forward | GCGCGCTTATTGCTTAAGAATAC |
| miR-137-3p Reverse | GTGCAGGGTCCGAGGT |
| U6 Forward | CTCGCTTCGGCAGCACA |
| U6 Reverse | AACGCTTCACGAATTTGCGT |
| KLF4 Forward | ACCTACACAAAGAGTTCCCATC |
| KLF4 Reverse | TGTGTTTACGGTAGTGCCTG |
| miR-137-3p mimics | UUAUUGCUUAAGAAUACGCGUAG |
| mimics-NC | UUGUACUACACAAAAGUACUG |
| KLF4-si1 | GGACGGCUGUGGAUGGAAATT |
| KLF4-si2 | GAGAGAGACCGAGGAGUUCTT |
| KLF4-si3 | AUCCUUCCUGCCCGAUCAGTT |
| KLF4-siNC | UUCUCCGAACGUGUCACGUTT |
| miR-137-3p FISH probe | CTACGCGTATTCTTAAGCAATAA |
| UPF1 Forward | CTGCAACGGACGTGGAAATAC |
| UPF1 Reverse | ACAGCCGCAGTTGTAGCAC |
