# Supplementary material for: CircUGP2 inhibits tumor progression via PI3K/AKT/mTOR signaling through sponging miR-137-3p and interacting with UPF1 in hepatocellular carcinoma
Source: Front Immunol. 2026 Oct 2;17:1926747. doi: 10.3389/fimmu.2026.1926747 (PMC13672610; doi:10.3389/fimmu.2026.1926747)
Supplement: Supplementary file 3 [file Table3.docx]

**Table S3.** Antibodies used in this study.

| Antibodies | Company |
| --- | --- |
| Anti-Ki-67 | Proteintech (27309-1-AP) |
| Anti-Flag | GenScript (A00187) |
| Anti-AGO2 | Proteintech (67934-1-Ig) |
| Anti-GAPDH | HUABIO (ET1601-4) |
| Anti-KLF4 | Abcam (ab215036) |
| Anti-PI3K | Abmart (T40115) |
| Anti-p-PI3K | Abmart (T40116) |
| Anti-AKT | Abcam (ab32505) |
| Anti-p-AKT | Abcam (ab81283) |
| Anti-mTOR | Abcam (ab32028) |
| Anti-p-mTOR | Abcam (ab109268) |
| Anti-UPF1 | Abcam (ab109363) |
